# Photoactivatable nanoCRISPR/Cas9 System Based on crRNA Reversibly Immobilized on Carbon Nanoparticles

**DOI:** 10.3390/ijms222010919

**Published:** 2021-10-09

**Authors:** Olga Semikolenova, Lubov Sakovina, Elizaveta Akhmetova, Daria Kim, Ivan Vokhtantsev, Victor Golyshev, Mariya Vorobyeva, Sergey Novopashin, Darya Novopashina

**Affiliations:** 1 Institute of Chemical Biology and Fundamental Medicine SB RAS, 630090 Novosibirsk, Russia; o.semikolenova@alumni.nsu.ru (O.S.); kodi99@list.ru (L.S.); kim.daria.nsk@gmail.com (D.K.); ivanvohtancev@gmail.com (I.V.); golyshevvictor@gmail.com (V.G.); maria.vorobjeva@gmail.com (M.V.); 2 Scientific Center of Genetic and Life Sciences, Sirius University of Science and Technology, 354000 Sochi, Russia; 3 Novosibirsk State University, 630090 Novosibirsk, Russia; Liza.khabardina@mail.ru; 4 Kutateladze Institute of Thermophysics SB RAS, 630090 Novosibirsk, Russia; sergeynovopashin@gmail.com

**Keywords:** CRISPR/Cas9 gene editing system, photocleavable oligonucleotides, carbon nanoparticles, magnetic nanoparticles, crRNA immobilization, pyrene, photoactivation

## Abstract

Here, we proposed a new approach to engineering a photoactivatable CRISPR/Cas9 gene-editing system. The novel nanoCRISPR/Cas9 system is based on the use of auxiliary photocleavable oligodeoxyribonucleotides (PC-DNAs) complementary to crRNA. PC-DNAs contained up to three UV-sensitive linkers made of 1-(2-nitrophenyl)-1,2-ethanediol inside the oligonucleotide chain. Immobilizing PC-DNAs on the surface of carbon nanoparticles through 3′-terminal pyrene residue provided sufficient blocking of crRNA (and corresponding Cas9 activity) before UV irradiation and allows for crRNA release after UV irradiation at 365 nm, which restores Cas9 activity. We optimized the length of blocking photocleavable oligonucleotide, number of linkers, time of irradiation, and the type of carbon nanoparticles. Based on the results, we consider the nanoCRISPR/Cas9 system involving carbon-encapsulated iron nanoparticles the most promising. It provides the greatest difference of functional activity before/after irradiation and can be used in prospective for magnetic field-controlled delivery of CRISPR system into the target cells or tissues and spatiotemporal gene editing induced by UV irradiation.

## 1. Introduction

An adaptive immune system of bacteria includes clustered regularly interspaced short palindromic repeats (CRISPR) and Cas proteins [1,2]. These elements provide protection against pathogenic phage infections. The main actors of CRISPR/Cas systems are CRISPR RNAs that recruit a Cas nuclease to complementary DNA or RNA for subsequent specific cleavage. The CRISPR/Cas9 system was proposed as a targetable nuclease for gene editing [3,4].

Inducible CRISPR/Cas9 systems have been developed to improve the spatiotemporal resolution of DNA cleavage [5,6]. For controlling the gene-editing system, two main approaches were developed. One approach is based on modified light-triggered Cas9 protein [7,8,9]. The second one means the usage of photocaged CRISPR RNA (crRNA or gRNA) [10,11,12,13] or an auxiliary photoblocking oligodeoxyribonucleotide complementary to CRISPR RNA [14,15].

The design of nucleic acids constructions containing photosensitive residues or linkers permits creating a spatiotemporally regulated system. Photocaged oligonucleotides have been used for the photoactivation of CRISPR/Cas9 gene-editing systems [5,14]. We supposed that the concept of a photoactivatable gene editing system can be implemented in an alternative manner, through reversible immobilization of the guide RNA on the surface of carbon nanoparticles.

Successful genome editing requires the effective delivery of the CRISPR/Cas9 system components into cells. Although viral vectors exhibit good gene transfection efficiency, they have drawbacks, such as possible carcinogenesis, mutagenesis, or immunogenicity complications. The direct delivery of the CRISPR/Cas9 system components as a ribonucleoprotein (RNP) complex has several advantages over the delivery of the corresponding gene constructions. There is no risk of spontaneous genome integration, timely degradation reduces potential off-target effects, and the whole system is ready for immediate use and does not need additional transcription and translation steps. Different approaches have been proposed for direct delivery of the gRNA/Cas9 complexes, including cell-penetrating peptides, metal and carbon nanoparticles, polymeric systems, and lipid nanoparticles [16,17,18,19,20,21,22]. Cell-penetrating peptides (CPP) recommended themselves as excellent tools capable of crossing the membrane to deliver therapeutic oligonucleotides inside the cells [23,24].

At the moment, a huge diversity of nanoparticles has been also proposed for cell delivery of nucleic acids [25]. The spectra of characteristics of nanoparticles such as surface type, size, charge, etc. are very wide. Therefore, a choice of concrete nanoparticle type is determined by the specific research task of the certain study. 

The unique physical and chemical properties of carbon nanotubes (CNTs) made them attractive as novel potential delivery vehicles for drugs and gene constructs [26,27]. CNTs include single-wall carbon nanotubes (SWCNTs), single graphene sheets rolled as cylindrical tubes, and multi-walled carbon nanotubes (MWCNTs), multi-layers of graphene sheets wrapped on each other in a cylindrical shape. Surface functionalization of SWCNTs has prompted their use to deliver plasmid DNA, microRNA, antisense oligonucleotides, and small interfering RNA (siRNA) to the target cells. Various methods have been developed for the immobilization of nucleic acids on the SWCNT surface. An effective approach for immobilizing nucleic acids on SWCNTs involves using pyrene as an anchor group strongly attaching to carbon surface through π−π-stacking interactions [28]. Non-modified SWCNTs possess low solubility and are poorly disperse in the aqueous solutions. Chemical modification, such as oxidation of SWCNTs, improves their solubilization in water and makes them suitable for biomedical applications [29]. 

Another promising drug delivery approach uses metal-containing nanoparticles, which possess a strong magnetic moment [30,31]. This approach is realized in magnetic drug targeting [32,33]. Different types of magnetic nanomaterials can be employed as magnetic drug delivery vehicles. One of the prospective variants of magnetic nanoparticles is carbon encapsulated iron nanoparticles (CEINP) with superparamagnetic properties.

SWCNT and CEINP look especially attractive for the creation of a photoactivatable CRISPR/Cas9 system. First, synthetic nucleic acids can be readily immobilized on their surface non-covalently by means of terminal pyrene modification. Second, surface-immobilized crRNA will be protected from nuclease digestion [34]. Moreover, surface immobilization also prevents RNA from interaction with other biomolecules, thus turning off its functional activity. Nanoparticles of this type also showed good potential as cell delivery vehicles and low toxicity, which is important for subsequent in vivo experiments. This combination of properties inspired us to propose a novel photoregulatable nanoCRISPR/Cas9 gene-editing system, which turns on after UV irradiation. To this purpose, we suggested immobilizing crRNA through the photocleavable oligodeoxyribonucleotide (PC-DNA) on the carbon nanoparticles (CNP) surface.

## 2. Results and Discussion

We aimed to engineer the system for targeted DNA cleavage so that it is inactive in the absence of UV light and readily switches on after UV irradiation. NanoCRISPR/Cas9 system suggested in our work comprises Cas9 protein, tracrRNA, and crRNA immobilized on carbon nanoparticles through 3′-pyrene modified photocleavable oligodeoxyribonucleotide (PC-DNA). In the ‘switched-off’ state, crRNA is blocked in complementary complex with PC-DNA immobilized on CNPs surface. UV irradiation destroys PC-DNA and breaks the complex, so crRNA releases from the CNP surface and induces Cas9-mediated cleavage of the target DNA.

The sequence of blocking oligodeoxyribonucleotide was chosen complementary to the fragment of guide RNA in the region of the target DNA binding, by analogy with [15]. We synthesized the series of PC-DNAs, which contained up to three photocleavable linkers inside the chain and the pyrene residue at the 3′-terminus (Table 1 and Table S1). 

We synthesized the phosphoramidite of 1-(2-nitrophenyl)-1,2-ethanediol (PL) and used it to prepare a series of photocleavable oligodeoxyribonucleotides by solid-phase phosphoramidite method, by analogy with [14]. Oligodeoxyribonucleotides contained two or three cleavable linkers. Unmodified oligodeoxyribonucleotides of the same sequence without the linkers served as controls.

**Figure 1:**
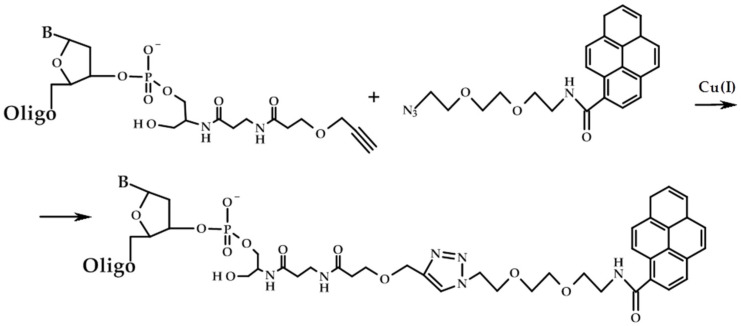
Scheme of the synthesis of 3′-pyrene conjugates.

The pyrene residue was attached to the 3′-alkyne modified oligodeoxyribonucleotides using the Cu-catalyzed cycloaddition method (also known as click-chemistry) (Figure 1). To this purpose, we used the modified CPG support containing the alkyne group, which gave 3′-alkyne modified oligonucleotides.

The pair of guide RNAs for the CRISPR/Cas9 system, crRNA, and tracrRNA, were also chemically synthesized by the same solid-phase phosphoramidite method. 3′-Fluorescein modified crRNA has been prepared with special fluorescein-modified CPG support. All oligonucleotides and their conjugates were isolated by preparative polyacrylamide gel electrophoresis (PAGE).

We tested three types of carbon nanoparticles for surface immobilization of crRNA: non-modified single-walled carbon nanotubes (SWCNT), carboxylated single-walled carbon nanotubes (SWCNT-COOH), and carbon-encapsulated iron nanoparticles (CEINP). SWCNT and SWCNT-COOH carbon nanoparticles are well-investigated and show good potential as nucleic acids delivery agents [27]. They have been employed for delivery of siRNA [35,36,37,38], antisense oligonucleotides [39], and DNA [40,41]. Earlier we showed that the IC_50_ values for the SWCNT-oligonucleotide hybrids are not achieved even at hybrid concentrations of 100 mg/L [28]. Moreover, immobilization on the CNT surface protects nucleic acids from nuclease digestion [34]. These data give the strong foundation to suppose that in our case RNA immobilized on the NPs surface would increase its stability to the nuclease digestion. Otherwise, magnetic properties of CEINP allow using them in perspective as magnetic field-controlled vehicles for cell delivery of nucleic acids. No toxic effects were revealed up to CEINP concentration 1 mg/L on HEK293T cell line by XTT assay (D. Novopashina and L. Yarinich, unpublished data). 

We employed the pyrene residue as an anchor group to immobilize oligonucleotides on the surface of carbon nanoparticles, to obtain CNP—nucleic acids hybrids. Earlier, we described this approach to prepare hybrids of pyrene-modified oligonucleotides with different single-walled carbon nanotubes (SWCNTs) [28,42,43]. The method is relatively simple, provides a high density of CNP functionalization and low toxicity of the resulting hybrid constructions. 

The non-covalent hybrids of the pyrene-oligonucleotide conjugates with CNP were formed by the adsorption of pyrene residues onto the defectless sites of the carbon surface. Pyrene fluorescence intensity tends to decrease upon the increase of the CNP concentration due to the quenching of the fluorescence on the carbon surfaces [44]. The quenching of pyrene fluorescence upon adsorption on CNTs is static [45]; the same character of quenching is reported for pyrene anchor groups used for immobilization of other cargo molecules on the CNT surface [46,47].

**Figure 2:**
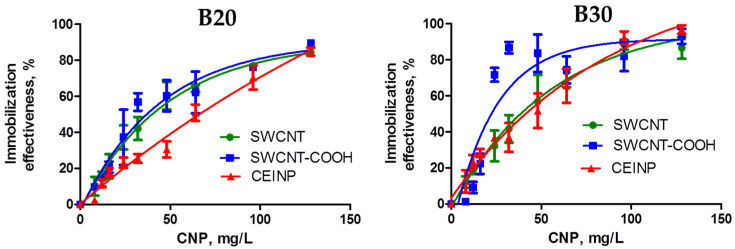
Isotherms of adsorption of oligonucleotides B20 and B30 on SWCNT, SWCNT-COOH, and CEINP surface. Conditions: 0.01 M phosphate buffer (pH 7.5), 1 µM oligonucleotides, detection of fluorescence at 380 nm.

We evaluated the efficiency of adsorption of the DNA conjugates on the CNT surface by pyrene fluorescence quenching [28]. The isotherms of adsorption for conjugates of the oligonucleotides B20 and B30 (Figure 2) show that both the oligonucleotide length and the nature of the CNP influence the adsorption.

The quantitative estimation of oligonucleotide adsorption capacities of CNPs showed higher values for 30-nt PC-DNA (B30_PL) compared to 20-nt PC-DNA (B20_PL). The maximal capacity (14 nmol/mg) was obtained for adsorption of B30_PL on the SWNT-COOH surface. The same oligonucleotide adsorbed on SWCNT and CEINP surfaces with twofold lower capacities (7.2 and 7.6 nmol/mg, correspondingly). The capacities of all three CNP types for adsorption of B20_PL were approx. 6.4 nmol/mg.

A comparative study of thermal stability of duplexes of crRNA with PC-DNAs or their non-modified photostable analogs revealed that the introduction of photocleavable linker reduces the thermal stability of duplexes, and increasing the number of linkers leads to further destabilization [14] (Table S3). Duplex dissociation constants obtained by gel-shift analysis correlated with the thermal stability of the corresponding duplexes (Table S3). The stability of duplexes of pyrene-modified B20_PL and B30_PL with crRNA_Flu were studied by gel-shift assay (Kd n.d. and 33.8 nM) and thermal denaturation method (28.8 and 44.7 °C), correspondingly. 

We also examined the cleavage of PC-DNA upon irradiation with 365 nm wavelength. The results show the complete cleavage of oligodeoxyribonucleotides after 5 min of irradiation (Figure S1), with slightly faster cleavage for PC-DNA with three photocleavable linkers. 

Several protocols are available for immobilizing the duplex on the CNP surface: (1) «mix-and-go», (2) algorithmic, and (3) hierarchal variant. The first one, the «mix-and-go» protocol, includes simultaneous mixing of all components (pyrene-modified oligonucleotide, crRNA, and CNP) with consequent hybrid formation. The second, algorithmic protocol, relies on duplex (pyrene-modified oligonucleotide and crRNA) formation in solution followed by its immobilization on the CNP surface. The last, hierarchal protocol requires immobilization of pyrene-modified oligonucleotide on a CNP surface and its subsequent hybridization with crRNA. We have previously tested all tree variants of duplex immobilization on the SWCNT surface (unpublished results) and demonstrated that the algorithmic variant is the most suitable for our goals.

**Figure 3:**
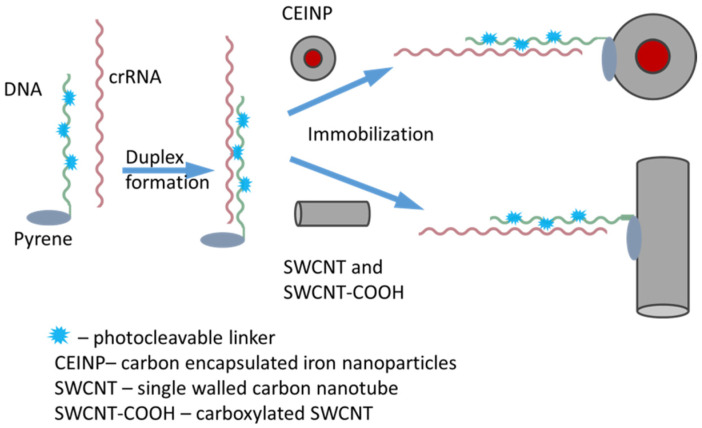
Scheme of the crRNA immobilization on the surface of carbon nanoparticles using auxiliary 3′-pyrene DNA conjugates.

Therefore, we used an algorithmic variant of immobilization of pre-formed duplex (B30_PL/crRNA or B20_PL/crRNA) on the CNP surface (Figure 3). First, we annealed crRNA_Flu or crRNA with the auxiliary photocleavable pyrene-bearing oligodeoxyribonucleotide. Then, pyrene-containing duplexes were immobilized on the CNP upon sonification and incubation at + 4 °C for 16 h.

**Figure 4:**
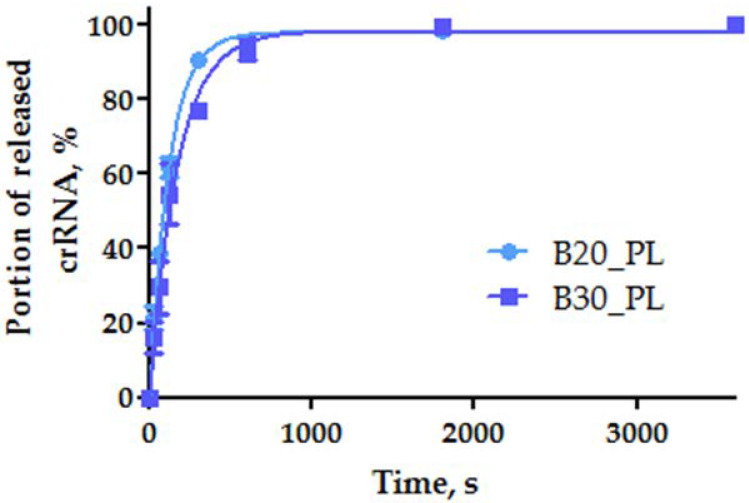
The effectiveness of releasing fluorescent crRNA_Flu immobilized on SWCNT surface through the auxiliary pyrene-modified PC-DNA upon UV irradiation (365 nm).

Next, we investigated the release of fluorescent crRNA_Flu from the CNP surface upon UV irradiation. The amount of free RNA was examined by PAGE analysis (Figure S2). It was shown that crRNA leaves the CNP surface quite quickly, with a half-release time of about 45 sec. Typical kinetic curves of RNA release during UV irradiation are given in Figure 4.

**Figure 5:**
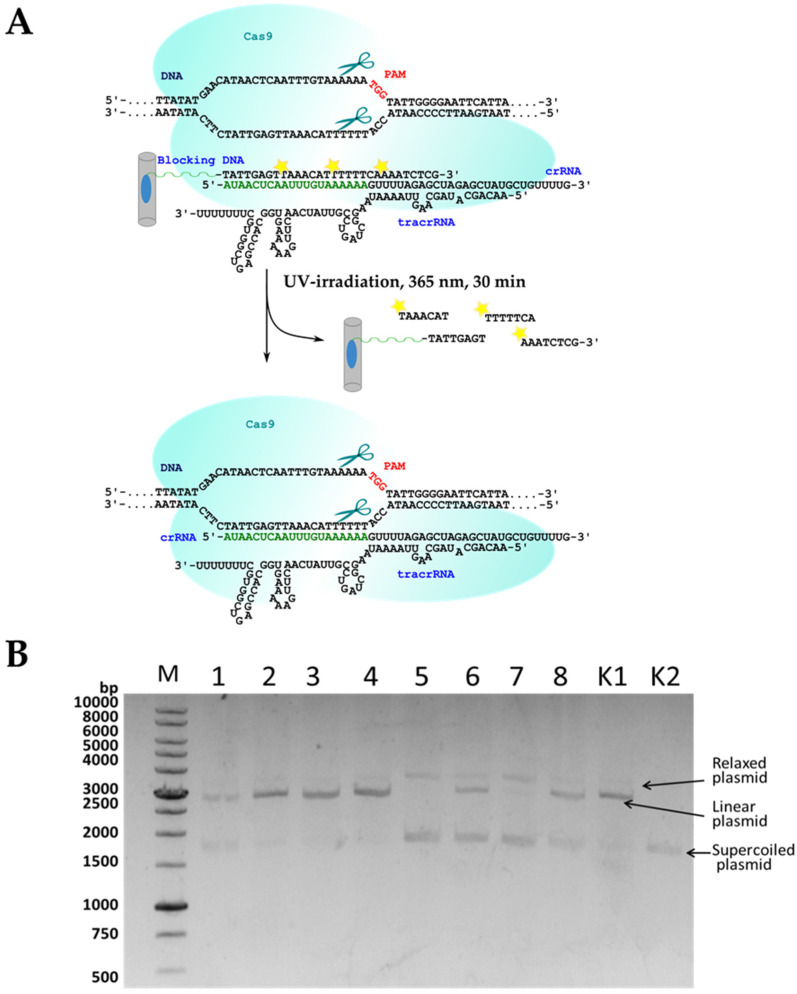
(**A**) The proposed mechanism of action of nanoCRISPR/Cas9 system. (**B**) Electropherogram of the cleavage products of the target DNA (a relaxed form of the plasmid that contained one nick, i.e., single-stranded break, and a linear form of the plasmid that contained a double-stranded break) after the action of crRNA/tracrRNA/Cas9. K1, the products of the cleavage of the target DNA with the Cas9 protein in the presence of tracrRNA and non-immobilized crRNAs, K2, initial target DNA plasmid, M, DNA marker. Cleavage of the plasmid supercoiled DNA target with Cas9 protein in the presence of tracrRNA and immobilized crRNA on SWCNT (lines 1, 5), SWCNT-COOH (lines 2, 6) or CEINP (lines 3, 7) or non-immobilized crRNA/B30_PL duplex (lines 4, 8) before (lines 5–8) and after (lines 1–4) irradiation at 365 nm. Conditions: 20 mM HEPES (pH 7.5), 100 mM KCl, 1 mM DTT, 0.5 mM Na_2_EDTA, 2 mM MgCl_2_, 5% glycerol. Cleavage of pBS2SKM by nanoCRISPR/Cas9 systems was performed at 37 °C for 1 h. Reaction mixtures were analyzed in agarose gel-electrophoresis.

We tested the functional activity of CNP-immobilized crRNAs in the model system, also containing tracrRNA, Cas9 nuclease, and DNA target. We called this system nanoCRISPR/Cas9 since it comprises nanoparticles as an essential component. The proposed mechanism of action of photo-regulated nanoCRISPR/Cas9 is presented in Figure 5a. The cleavage of supercoiled plasmid DNA by the Cas9 protein in the presence of guide RNAs gave a mostly linear form of the plasmid and a small amount of a relaxed form that contained one nick (a single-stranded break) (Figure 5b). Earlier, we found that 30-nt oligomer B30 blocks the activity of the CRISPR system better than B20, which nicely coincides with the stability of their duplexes with crRNA [14]. That is why we chose B30_PL for immobilization of crRNA in the nanoCRISPR/Cas9 system. The experiments on the plasmid DNA cleavage by the combination of Cas9 nuclease, tracrRNA, and CNP-immobilized crRNAs showed that immobilization on SWCNT, and CEINP surface sufficiently blocks the cleavage.

**Figure 6:**
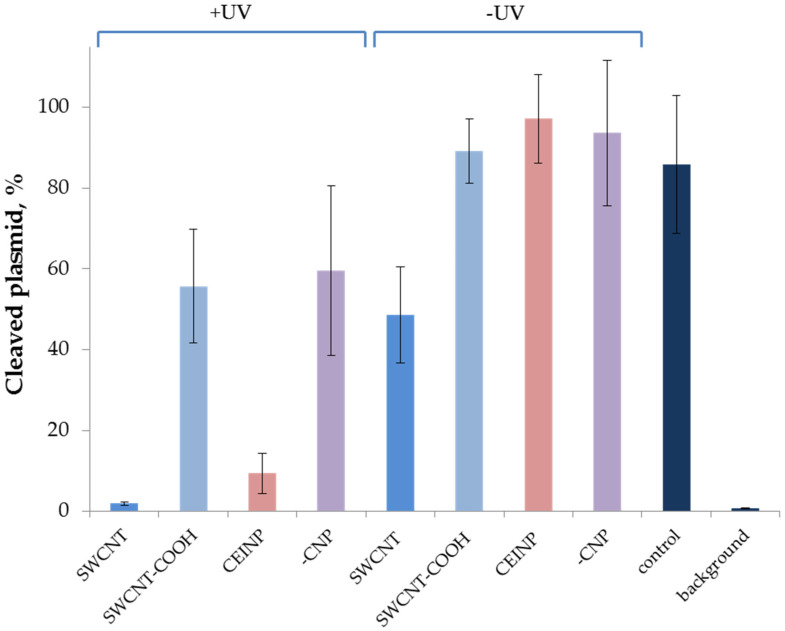
DNA plasmid cleavage by nanoCRISPR/Cas9 systems with CNP-immobilized (SWCNT, SWCNT-COOH, CEINP) with or without UV irradiation. crRNA immobilized on the surface using the PC-DNA (B30_PL). The plasmid cleavage was performed with and without UV irradiation. “Control” corresponds to the DNA cleavage by crRNA/tracrRNA/Cas9 in the absence of PC-DNA and CNPs. “Background” corresponds to the cleavage extent in the absence of nuclease, “-CNP”-to the non-immobilized duplex of PC-DNA (B30_PL) with crRNA. Conditions: crRNA immobilized through of B30_PL, the temperature of cleavage reaction was 37 °C, irradiation 30 min at 365 nm.

Otherwise, UV irradiation led to the activation of the nanoCRISPR/Cas9 system, and we observed a strong increase in the dsDNA cleavage efficiency (Figure 5 and Figure 6). Exposure to light at 365 nm for 30 min made it possible to restore the activity of the CRISPR system for SWCNT-COOH and CEINP or increase the effectiveness of cleavage for SWCNT. The nanoCRISPR/Cas9 system on the base of CEINP is the most promising due to the most remarkable activity difference before and after irradiation and the possibility of magnetic field-controlled delivery of the CRISPR system into the targeted tissue and cells.

## 3. Materials and Methods

### 3.1. Materials

A controlled pore glass support (CPG) derivatized with 2′-O-TBDMS-G, 2′-O-TBDMS-U, deoxycytosine (dC) or deoxythymidine (dT), 5′,N-protected 2′-O-TBDMS-ribo (A, C, G or U) and deoxyribo (dA, dC, dG or dT,) phosphoramidites, 3′-alkyne-modifier serinol CPG and 3′-(6-fluorescein) CPG were purchased from Glen Research Inc (Sterling, VA, USA). Sodium perchlorate, 40% aqueous methylamine solution, and *N*-methylimidazole were purchased from Acros Organics (Fair Lawn, NJ, USA,); 4,4′-dimethoxytrityl chloride was purchased from Alfa Aesar (Lancashire, UK); Stains-all dye, ammonium persulfate, dichloroacetic acid, acrylamide, *N,N′*-methylenebisacrylamide, 2,6-lutidine, ethidium bromide, and tris(hydroxymethyl)aminomethane were purchased from Fluka (Buchs, Switzerland); xylene cyanol FF, bromophenol blue, and *N,N,N′,N′*-ethylenediamine tetraacetic acid were purchased from Serva (Heidelberg, Germany); 1-(2-nitrophenyl)-1,2-ethanediol, trimethylamine, 5-ethylthio-1H-tetrazole, trimethylamine trihydrofluoride, ethoxytrimethylsilane, single-walled carbon nanotubes, and 2-cyanoethyl-*N,N,N′,N′*-tetraisopropyl phosphoramidite were purchased from Sigma-Aldrich (USA). Ascorbic acid, 10 mM Cu(II)-TBTA Stock in 55% DMSO, and pyrene azide were purchased from Lumiprobe (Moscow, Russia). All solvents (THF, DMSO, CH_3_CN, ethanol, CH_2_Cl_2_ (various vendors)) were dried by 3 Å molecular sieves or by distillation and stored over CaH_2_. Kieselgel F254 thin-layer chromatography plates were purchased from Merck (Kenilworth, NJ, USA). 

Recombinant Cas9 endonuclease and pBS2SKM Psp2 TTG plasmid based on the pBluescript II SK(-) vector that contained the insert of the protospacer and PAM (5′-TGG-3′) were obtained according to the standard protocol [48].

### 3.2. Equipment 

All aqueous solutions were prepared using deionized water (Millipore Simplicity System; Millipore, Burlington, Mass, USA). Oligonucleotide solutions were concentrated on a vacuum Concentrator Plus (Eppendorf, Hamburg, Germany). Oligonucleotides were precipitated and centrifuged on MiniSpin Plus centrifuges (Eppendorf, Hamburg, Germany). The solutions were stirred on a Thermomixer Comfort (Eppendorf, Hamburg, Germany). The optical absorption of oligonucleotide solutions was measured on a NanoDrop 1000 spectrophotometer (Thermo Fisher Scientific, Waltham, MA, USA). 

### 3.3. Physical Measurements

^1^H-NMR and ^31^P-NMR spectra of the compounds were measured with CDCl_3_ as a solvent, using AVANCE III 400 NMR spectrometer (Bruker Corporation, Billerica, MA, USA).

Mass spectra were recorded using a MALDI-TOF Autoflex Speed mass spectrometer (Bruker Daltonics, Billerica, MA, USA). 

The optical densities of the solutions of oligonucleotides and their conjugates were measured using a NanoDrop 1000 spectrophotometer (Thermo Fisher Scientific, Waltham, MA, USA). To determine molar concentrations of the oligonucleotides and their conjugates, we used corresponding molar extinction coefficients at 260 nm calculated by IDT OligoAnalyzer™ Tool. 

### 3.4. Synthesis of 2-O-4,4′-dimethoxytrityl-1-(2-nitrophenyl)-1,2-ethanediol (I)

Synthesis of 2-O-4,4′-dimethoxytrityl-1-(2-nitrophenyl)-1,2-ethanediol (I) was carried out by analogy with [49,50] with some modifications. The solution of 4,4′-dimethoxytrityl chloride (406.6 mg, 1.2 mmol) in dry pyridine (3 mL) was added to the solution of 1-(2-nitrophenyl)-1,2-ethanediol (200 mg, 1.1 mmol) in dry pyridine (1 mL), and the mixture was kept for 1 h. TLC in the system of solvents (ethyl acetate/hexane, 1:4) was used to control the reaction. After completion of the reaction, methanol (0.5 mL) was added to decompose the excess of 4,4′-dimethoxytrityl chloride. The reaction mixture was evaporated, and the residue was dissolved in methylene chloride, followed by extraction with a saturated solution of NaHCO_3_. The combined organic layers were dried over anhydrous Na_2_SO_4_ and evaporated to dryness. The product was isolated by column silica gel chromatography using a concentration gradient of ethyl acetate in hexane (0–35%). R_f_ 0.11. The yield of the product was 85%. ^1^H NMR (500 MHz, CDCl_3_) δ, ppm: 7.85 (d, 1H), 7.82 (d, 1H), 7.62 (t, 1H), 7.42–7.35 (m, 3H), 7.30–7.16 (m, 11H), 6.78 (m, 4H), 5.50 (dd, 1H), 3.78 (s, 7H), 3.62 (dd, 1H), 3.16 (dd, 1H).

### 3.5. Synthesis of 1-(2-cyano-N,N-diisopropyl-phosphoramidite)-2-O-4,4′-dimethoxytrityl-1-(2-nitrophenyl1,2-ethanediol (II)

Synthesis of 1-(2-cyano-N,N-diisopropyl-phosphoramidite)- 2-O-4,4′-dimethoxytrityl-1-(2-nitrophenyl1,2-ethanediol (II) was carried out by analogy with [49] with some modifications. 5-Ethylthiotetrazole (195 mg) in abs. acetonitrile (1.2 mL) and N,N-diisopropylethylamine (300 mL) were added to the solution of compound (I) (480 mg, 1 mmol) in abs. acetonitrile (3 mL), followed by the addition of 2-cyanoethyl-N,N,N′,N′-tetraisopropyl phosphoramidite (625 μL, 2 mmol). TLC in the system of solvents (chloroform-hexane-ethyl acetate, 4:4:2) was used to control the reaction. After completion of the reaction, ethyl acetate (6 mL) was added. The resulting mixture was filtered, and the precipitate was washed several times with ethyl acetate. The filtrate was extracted with a saturated solution of NaHCO_3_, and the combined organic layers were dried over anhydrous Na_2_SO_4_, filtered, and evaporated to dryness. The product was isolated by column silica gel chromatography using a concentration gradient of ethyl acetate in hexane (0–30%). R_f_ 0.80. The yield of the product was 61%. ^1^H NMR (300 MHz, CDCl_3_) δ, ppm: 7.89 (d, 1H), 7.80–7.76 (d, 1H), 7.59–7.51 (t, 2H), 7.41–7.33 (m, 3H), 7.29–7.10 (m, 13H), 6.80–6.72 (m, 5H), 5.81–5.72 (m, 1H), 3.90–3.80 (m, 10H), 3.56–3.38 (m, 3H), 3.27–3.20 (m, 1H), 2.63–2.38 (m, 2H), 2.04–1.02 (m, 1H), 1.28–1.11 (m, 12H), 0.94–0.80 (d, 7H). ^31^P-NMR (150 MHz, CDCl_3_) δ, 150.1 ppm.

### 3.6. Phosphoramidite Synthesis of Oligonucleotides

Oligonucleotides were synthesized according to the optimized synthetic protocol on an automated ASM-800 DNA/RNA synthesizer (Biosset, Novosibirsk, Russia). The coupling reaction with phosphoramidite (**II**) (0.1 M solution in abs. acetonitrile) occurred for 30 min. Oligonucleotides were deprotected and removed from the polymer support by the treatment with 40% methylamine for 15 min at 65 °C with stirring or, in the case of 3′-fluorescein containing oligonucleotides, with 30% aqueous ammonia at room temperature for 16 h. In the case of oligoribonucleotides, the 2′-O-TBDMSi protecting groups were removed by a freshly prepared NMP-Et_3_N-Et_3_N … 3HF mixture (1.5:0.75:1, *v*/*v*/*v*) at 65 °C with stirring for 1.5 h, followed by ethoxytrimethylsilane treatment and precipitation of oligoribonucleotides with diethyl ether.

### 3.7. Synthesis of 3′-Pyrene Conjugates

Triethylammonium acetate buffer (pH 7.0), 10 mM pyrene azide (Lumiprobe, Moscow, Russia) in DMSO, 5 mM ascorbic acid solution in water, and 10 mM Cu(II)-TBTA stock in 55% DMSO were added to the water solution of 3′-alkyne-modified oligodeoxyribonucleotide (25 nmol) according to the protocol of click reagent supplier (Lumiprobe, Moscow, Russia). The reaction mixture was incubated at room temperature overnight. Oligonucleotide conjugates were precipitated with 2% NaClO_4_ in acetone and washed with acetone. The pellets were dried in air, dissolved in water, and analyzed by gel electrophoresis. The conversion of the oligonucleotide to the conjugate was almost quantitative, according to the PAGE data. The structures of conjugates were confirmed by electronic absorption spectra and fluorescence spectra. 

### 3.8. Purification of Oligonucleotides and Their Conjugates

Deprotected oligonucleotides and 3′-pyrene conjugates were isolated by 15% denaturing polyacrylamide gel electrophoresis (PAGE) in the 0.4 mm gel, followed by elution from the gel with 0.3 M NaClO_4_ solution, desalted with Sep-Pak C18 cartridge (Waters, USA), and precipitated as sodium salts. Oligonucleotides in the gel were visualized when the gel was applied to a DC-Alufolien Kieselgel 60 F254 plate (Merck, Darmstadt, Germany) in the light of a UV lamp (λ = 254 nm). To avoid the cleavage reaction under the UV radiation in the case of PC-DNA, the main part of the gel that contained the product was covered with glass, thus leaving only a small part to be sufficient for visualization. crRNA, crRNA_Flu, and tracrRNA were eluted from the gel with 0.3 M sodium acetate solution, pH 5.2, followed by ethanol precipitation. 

### 3.9. Oxidation of Carbon Nanotubes

Raw single-walled carbon nanotubes (Sigma, St. Louis, MO, USA) were oxidized as described in [51]. Briefly, 500 mg raw SWCNTs were dispersed in 200 mL 70% nitric acid and heated at 115 °C for 6 h. SWCNT-COOH were recovered by filtration using PTFE filters with a pore diameter of 100 nm (Millipore, Burlington, Mass, USA), rinsed thoroughly with deionized water, and dried. The substance was characterized as in [43]. 

### 3.10. Carbon Encapsulated Iron Nanoparticles Preparation

We used the plasma-arc method for carbon encapsulated iron nanoparticles preparation as described in [52,53]. Briefly, the experiments were carried out in the direct current (DC) electric arc with the current of 100 A in the buffer gas (helium) at 50 Torr. The spray electrode (anode) was a graphite rod 70 mm in length and 7 mm in diameter. A hole (with a diameter of 4 mm) was drilled in the center of the electrode to be filled with the graphite-iron mixture powder. The Fe/C weight ratio was 2/1. The CEINPs were precipitated on a cooled shield located 5 cm away from the arc discharge area. The obtained CEINPs were characterized by high-resolution transmission electron microscopy (TEM) using the JEM-2010 electron microscope (JEOL, Tokyo, Japan), local energy dispersive X-ray analysis (EDXA) using the EDX spectrometer (EDAX Co.), XRD analysis using the Bruker D8 Advance diffractometer, equipped with the Lynxeye (1D) linear detector, and magnetization analysis using the magnetometer SM-150L ZH Instrument, and SQUID magnetometer MPMSXL (Quantum Design) (Figure S3).

### 3.11. Adsorption Isotherms of Pyrene Conjugates of Oligonucleotides onto SWCNTs

To obtain solutions of the hybrids, CNP (SWCNT, SWCNT-COOH, or CEINP) were added to a buffered (10 mM Na phosphate buffer, pH 7.5) oligonucleotide solution (1 μM) to a final concentration of 1–128 mg L^−1^ and sonicated for 30 min using Sonorex Super RK 31 H ultrasonic bath (Bandelin Electronic, Berlin, Germany). The fluorescence spectra of samples were recorded upon excitation at 345 nm using Cary Eclipse Fluorescence Spectrophotometer (Varian Inc., Palo Alto, CA, USA) at 25 °C with the use of a 4 mm quartz cuvette. The amount of oligonucleotide adsorbed onto the CNPs was calculated as Equation (1)

(1) $$\left(1-\frac{I_{Pyr}}{I_{Pyr}^{0}}\right)\cdot 100\%$$

where $I_{Pyr}$ is the intensity of the pyrene fluorescence at 378 nm in the oligonucleotide—SWCNT hybrid solution, and $I{\;}_{Pyr}^{0}\;$ is the intensity of the pyrene residue fluorescence at 378 nm in the reference oligonucleotide solution at the same concentration (1 μM). For calculations, stable dispersions of carbon nanoparticles were considered as solutions.

The capacities of CNPs for the adsorption of oligonucleotide were estimated by analogy with [28] in the first point of the saturation plateau in adsorption isotherms as Equation (2)

(2) $$\frac{C_{ON}\cdot n_{ads}}{C_{CNP}}$$

where $C_{ON}$ is the total oligonucleotide concentration (1 μM), $n_{ads}$ is the amount of the adsorbed oligonucleotide [%] in a given point as estimated from an isotherm, CNP is the concentration of the CNP [mg L^−1^] in the same point.

### 3.12. Immobilization of crRNA on CNP Surface

Hybrids were obtained using the algorithmic method. The solution containing the auxiliary oligodeoxyribonucleotide (B20, B30, B20_PL or B30_PL) and crRNA or crRNA_Flu in a concentration of 1 μM and the components of buffer (10 mM Na phosphate buffer, pH 7.5 or 100 mM NaOAc, 2 mM Mg(OAc)_2_, 30 mM HEPES-KOH (pH 7.5)) were incubated at 95 °C for 2 min and slowly cooled to the room temperature. The solution of 10 mg/L CNP in ultrapure water was added to the preformed duplex with the final volume of 10 or 50 µL. The samples were sonicated for 30 min and incubated at 4 °C overnight. 

### 3.13. The Release of Fluorescent crRNA_Flu Immobilized on CNP Surface

The CNP-immobilized duplexes of PC-DNA with crRNA_Flu were irradiated with light at 365 nm for 0.5, 1, 2, 5, 10, 15, 30, and 60 min. The cleavage products were analyzed in denaturing 15% PAGE, followed by visualization using the E-Box-CX5 gel documentation system (Vilber Lourmat, France). The images were quantified using the Quantity One program (Bio-Rad, United States). The portion of the released crRNA_Flu was calculated using the Microsoft Excel software. Non-immobilized crRNA_Flu taken at the same quantity served as a control corresponding to the 100% of free RNA. The parameters were calculated in the GraphPad Prism 5.0.4.533 software package (Graph-Pad, United States) using the Equation (3):

(3) $$f_{a}=P_{st}\;\cdot \left(1-e^{k_{1}t}\right)$$

where $f_{a}$ is a portion of the released crRNA_Flu; $P_{st}$ is a portion of the released crRNA_Flu during the transition of the reaction to the stationary phase (the maximum degree of release); $k_{1}$ is the pseudo-first-order reaction constant; *t* is the reaction time. 

### 3.14. Cleavage of Plasmid DNA by the Cas9 Protein in the Presence of Immobilized Guide RNAs

The reactions were carried out in the cleavage buffer (10 μL) containing 20 mM HEPES (pH 7.5), 100 mM KCl, 1 mM dithiothreitol, 0.5 mM Na_2_EDTA, 2 mM MgCl_2_, and 5% glycerol. The control solution contained all components except RNAs and Cas9 protein. Initially, the effector complex was assembled. For this purpose, we prepared the solutions containing either immobilized or non-immobilized crRNA (1 μM, 1.35 μL, 1.35 pM), tracrRNA (1 μM, 1.35 μL, 1.35 pM, each) and the Cas9 protein (2.24 μM, 0.602 μL, 1.35 pM) in the cleavage buffer. The mixtures were vortexed and kept for 15 min at 37 °C. The plasmid pBS2SKM containing the protospacer and PAM sequence (5′-TGG-3′) (1 μL, 50 ng/mL, 27 fmol) was added to each vial. The reaction mixtures were stirred and kept for 1 h at 37 °C in the absence of direct sunlight. The reaction was stopped by adding the quenching buffer (2.5 μL) that contained 250 mM Na_2_EDTA, 1.2% SDS, 0.01% bromophenol blue, and 30% glycerol. The PC-DNAs (B20_PL or B30_PL) were deactivated by UV irradiation at 365 nm for 30 min.

### 3.15. The Efficiency of the Plasmid Cleavage

The cleavage of the supercoiled form of the plasmid to the relaxed and linear forms by the Cas9 protein in the presence of guide RNAs was analyzed by gel electrophoresis in 1% agarose gel in TAE buffer (4 mM Tris, 3 mM CH_3_COOH, 0.07 mM Na_2_EDTA) in the presence of 2.5 μL of ethidium bromide (10 mg/mL). The reaction mixture (10 μL) in quenching buffer was loaded to the gel. The 1 kb DNA Ladder (250–10,000 bp dsDNA fragments; SibEnzyme, Russia) was used as a reference to evaluate the mobility of the cleavage products. The products were visualized using the E-Box-CX5 gel documentation system (Vilber Lourmat, France). The images were quantified using the Quantity One program (Bio-Rad, United States). The portion of the plasmid cleavage was calculated by the following Equation (4):

(4) $$N_{\Sigma }=\left(I_{lin}+I_{rel}\right)\;\cdot 100\%/\left(I_{lin}+I_{rel}+\frac{I_{superc\;}}{k}\right)$$

where $N_{\Sigma }$ is the total plasmid cleavage; $I_{lin}$ is the intensity of the band corresponding to the linear form of the plasmid; $I_{rel}$ is the intensity of the band corresponding to the relaxed form of the plasmid; $I_{superc}$ is the intensity of the band corresponding to the supercoiled form of the plasmid; *k* = 1.14 is the coefficient of staining efficiency of the supercoiled form of DNA relative to the relaxed form [54].

## 4. Conclusions

In this study, we proposed and developed a new approach to CRISPR/Cas9-mediated spatiotemporal gene editing induced by UV irradiation and engineered a so-called nanoCRISPR/Cas9 system. The system bases on the photocleavable DNA oligonucleotide immobilized on the surface of carbon nanoparticles. This oligomer blocks the crRNA component of CRISPR/Cas9 by complementary interactions until UV irradiation. The irradiation at 365 nm destroys the photocleavable DNA, thus releasing crRNA for normal functioning of Cas9 nuclease and targeted DNA cleavage. We thoroughly tested the usability of this approach in the model system and tuned the key parameters such as the length of blocking DNA, number of photocleavable linkers inside the chain, and the type of carbon nanoparticles. It turned out that the nanoCRISPR/Cas9 system on the base of magnetic CEINP seems to be the most promising due to the biggest difference of functional activity in ‘off’ and ‘on’ states and also because of the possibility of magnetic field-controlled delivery of CRISPR system into the target cells and tissues.

Taken together, the obtained results allow considering the proposed nanoCRISPR/Cas9 system prospective for photocontrolled gene editing. To use this system in vivo, additional studies are required with particular attention to the features of genome editing in living cells.

## Figures and Tables

**Table 1:** Synthetic oligonucleotides used in the study: PC-DNAs and their 3′-pyrene containing conjugates, crRNA, and tracrRNA.

Name	Sequence, 5′-3′
B20	5′-TTTTTTACAAATTGAGTTATCC-*Pyr*
B20_PL	5′-TTTTTT-PL-ACAAA-PL-TTGAG-PL-TTATCC-*Pyr*
B30	5′-GCTCTAAAACTTTTTTACAAATTGAGTTAT-*Pyr*
B30_PL	5′-GCTCTAAA-PL-ACTTTTT-PL-TACAAAT-PL-TGAGTTAT-*Pyr*
crRNA_Flu	5′-AUAACUCAAUUUGUAAAAAAGUUUUAGAGCUAUGCUGUUUUG-*Flu*
crRNA	5′-AUAACUCAAUUUGUAAAAAAGUUUUAGAGCUAUGCUGUUUUG
tracrRNA	5′-AACAGCAUAGCAAGUUAAAAUAAGGCUAGUCCGUUAUCAACUUGAAAAAGUGGCACCGAGUCGGUGCUUUUUUU

PL—photocleavable linker on the base of 1-(2-nitrophenyl)-1,2-ethanediol; *Pyr*—pyrene residue; *Flu*—fluorescein residue.

## Supplementary Materials

The following are available online at https://www.mdpi.com/article/10.3390/ijms222010919/s1, Figure S1: Example of electrophoretic analysis of the cleavage of fluorescently labeled photomodified oligodeoxyribonucleotides and kinetic curves of their cleavage, Figure S2: The release of fluorescent crRNA_Flu immobilized on CNP surface through the auxiliary pyrene-modified PC-DNA upon UV irradiation, Figure S3: The CEINPs characterization, Table S1: PC-DNA and their 3′-pyrene containing conjugates, crRNA, and tracrRNA, Table S2: Molecular mass for oligonucleotides and their 3′-pyrene conjugates, Table S3: Dissociation constants and melting temperatures of the duplexes of PC-DNA and their analogs with crRNA_Flu.
