# Supplementary material for: Photoactivatable nanoCRISPR/Cas9 System Based on crRNA Reversibly Immobilized on Carbon Nanoparticles
Source: Int J Mol Sci. 2021 Oct 9;22(20):10919. doi: 10.3390/ijms222010919 (PMC8539621; doi:10.3390/ijms222010919)
Supplement: Supplementary file 1 [file ijms-22-10919-s001.zip › ijms-1400251-supplementary.pdf]

Supplementary Materials

Photoactivatable nanoCRISPR/Cas9 system

Olga Semikolenova [1,2], Lubov Sakovina [1,2], Elizaveta Akhmetova [3], Daria Kim [1,2], Ivan Vokhtantsev [1,3], Victor Golyshev [1], , Maryia Vorobyeva [1,2], Sergey Novopashin [4], Darya Novopashina[1,2,3]*

1 Institute of Chemical Biology and Fundamental Medicine SB RAS, Novosibirsk, Russia
2 Sirius University of Science and Technology, Sochi, Russia
3 Novosibirsk State University, Novosibirsk, Russia
4 Kutateladze Institute of Thermophysics SB RAS, Novosibirsk, Russia
* Correspondence: e-mail@e-mail.com; Tel.: (optional; include country code; if there are multiple corresponding authors, add author initials)

Table S1. PC-DNA and their 3'-pyrene containing conjugates, crRNA and tracrRNA.

Name	Sequence, 5'-3'
**B20_c**	5′-TTTTTTACAAATTGAGTTATCC
**B20_PL3_c**	5′-TTTTTT-**PL**-ACAAA-**PL**-TTGAG-**PL**-TTATCC
**B20**	5′-TTTTTTACAAATTGAGTTATCC-***Pyr***
**B20_PL**	5′-TTTTTT-**PL**-ACAAA-**PL**-TTGAG-**PL**-TTATCC-***Pyr***
**B30_c**	5′-GCTCTAAAACTTTTTTACAAATTGAGTTAT
**B30_PL2_c**	5′-GCTCTAAAAC-**PL**-TTTTTTACAA-**PL**-ATTGAGTTAT
**B30_PL3_c**	5′-GCTCTAAA-**PL**-ACTTTTT-**PL**-TACAAAT-**PL**-TGAGTTAT
**B30**	5′-GCTCTAAAACTTTTTTACAAATTGAGTTAT-***Pyr***
**B30_PL**	5′-GCTCTAAA-**PL**-ACTTTTT-**PL**-TACAAAT-**PL**-TGAGTTAT-***Pyr***
**crRNA_Flu**	5′-AUAACUCAAUUUGUAAAAAAGUUUUAGAGCUAUGCUGUUUUG-***Flu***
**crRNA**	5′-AUAACUCAAUUUGUAAAAAAGUUUUAGAGCUAUGCUGUUUUG
**tracrRNA**	5′-AACAGCAUAGCAAGUUAAAAUAAGGCUAGUCCGUUAUCAACUUG-AAAAAGUGGCACCGAGUCGGUGCUUUUUUU

PL – photocleavable linker on the base of 1-(2-nitrophenyl)-1,2-ethandiol; ***Pyr*** – pyrene residue; ***Flu*** – fluorescein residue.

Analysis of oligonucleotide homogeneity

The homogeneity of the synthesized oligonucleotides was confirmed by gel electrophoresis in 12% or 15% PAAG (acrylamide-N,N'-methylene bisacrylamide, 30 : 0.5 or 29 : 1, respectively) under denaturing conditions (8 M urea, 50 mM Tris-H_3BO_3, pH 8.3, 0.1 M Na_2EDTA). The samples were applied to the gel in 5 μL of 8 M urea that contained 0.025% xylene cyanol FF. Oligonucleotides were visualized on the gel with Stains-all dye (50 mg of Stains-all in 100 μL of a water–formamide mixture, 1 : 1). After staining, the gels were dried on a GelDryer 583 (Bio-Rad, United States).

Mass spectrometry of oligonucleotides and their 3'-pyrene conjugates

Mass spectra were recorded using a MALDI-TOF Autoflex Speed mass spectrometer (Bruker Daltonics, Billerica, MA, USA).

Table S2. Molecular mass for oligonucleotides and their 3'-pyrene conjugates.

Name	Sequences, 5'-3'	Molecular weight	
		Calculated	Found
**B20**	5'-TTTTTTACAAATTGAGTTATCC-***Pyr***	7137,85	7139,81
**B30**	5'-GCTCTAAAACTTTTTTACAAATTGAGTTAT-***Pyr***	9977,73	9972,68
**B20_c**	5'-TTTTTTACAAATTGAGTTATCC	6689,36	6690,34
**B30_c**	5'-GCTCTAAAACTTTTTTACAAATTGAGTTAT	9168,94	9169,02

Pyr – pyrene residue.

Kinetics of the cleavage of photomodified oligodeoxyribonucleotides under irradiation

Oligodeoxyribonucleotides that contained photocleavable linkers and the 5'-fluorescein residue (B30_PL and B30_PL) (10 μM solutions in water or buffer consisted of 20 mM HEPES, pH 7.5, 100 mM KCl, 1 mM dithiothreitol, 0.5 mM Na_2EDTA, 2 mM $MgCl_2$, and 5% glycerol) were irradiated with light at 365 nm for

1, 2, 5, 10, 15, and 30 min. The cleavage products were analyzed in denaturing 15% PAGE (Figure S1A), followed by visualization by UV irradiation at 312 nm on a Quantum Vilber Lourmat gel documentation system (Vilber Lourmat, France). The images were quantified using the Quantity One program (Bio-Rad, United States). The portion of the cleavage of the modified oligodeoxyribonucleotide was calculated using the Microsoft Excel software. The parameters were calculated in the GraphPad Prism 5.0.4.533 software package (Graph- Pad, United States) using the equation:

$$f_a = P_{st} \cdot (1 - e^{k_1 t}),$$

where f_a is a portion of the reaction product; P_{st} is a portion of the product during the transition of the reaction to the stationary phase (the maximum degree of cleavage); k_1 is the pseudo-first-order reaction constant; t is the reaction time (Figure S1B).

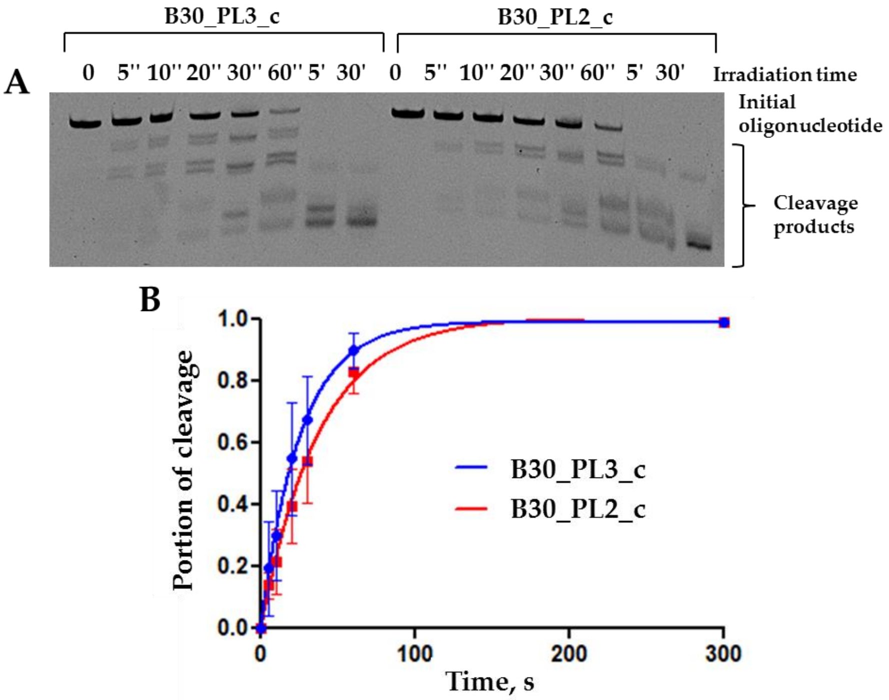

Figure S1. (**A**) Example of electrophoretic analysis of the cleavage of fluorescently labeled photomodified oligodeoxyribonucleotides B30_PL3_c and B30_PL2_c. (**B**) Kinetic curves of the cleavage of B30_PL3_c and B30_PL2_c.

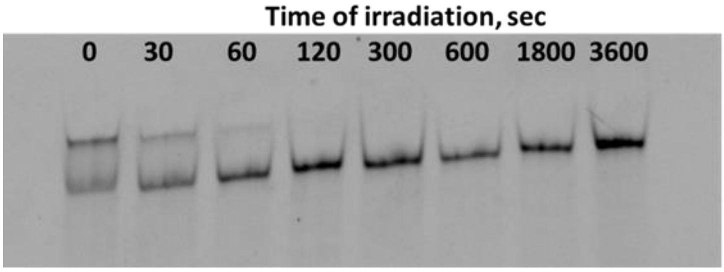

Figure S2. The release of fluorescent crRNA_Flu immobilized on SWCNT surface through the auxiliary pyrene-modified PC-DNA upon UV-irradiation. 15% denaturating PAGE, B30_PL/crRNA_Flu duplex concentration was $1 \cdot 10^{-6}$ M, temperature was 25°C, wavelength of irradiation was 365 nm.

Thermal denaturation of guide RNAs duplexes with blocking oligodeoxyribonucleotides

Differential curves of thermal denaturation of duplexes were recorded on a Cary 300 BioMelt spectrophotometer equipped with a temperature-controlled optical cuvette (Varian, Australia). The concentration of the oligonucleotide components was 1 μM in a buffer that contained 0.1 M NaCl, 10 mM sodium cacodylate (pH 7.4), and 1 mM Na_2EDTA. The temperature was changed at a rate of 0.5°C/min. Absorption was detected at two wavelengths (260 and 270 nm). Thermal denaturation and renaturation curves were processed using the Microsoft Office 2010 software. The melting points were evaluated as the maxima of the first derivatives of the melting curves relative to temperature (Table S3).

Dissociation constant determination by gel-shift analysis

Oligodeoxyribonucleotide (B20_c, B20_PL3_c, B30_c, B30_PL2_c, B30) in different concentrations (1, 3, 5, 7, 10, 20 or 3 μM) was heated in a buffer that contained 100 mM NaCl, 89 mM Tris-H_3BO_3, pH 8.3, 0.1% xylencyanol FF, 0.1% bromophenol and 30% glycerol, at 95°C for 2 min. Then the mixture was kept at 25°C for 15 min and crRNA was added to a concentration of 10 μM. The mixture was incubated at 25°C for 2 hours and was loaded on non-denaturing electrophoresis in 15% polyacrylamide gel. The resulting gels were visualized using Bio-Rad Gel Imagining System (BioRad, USA). The images were quantified using the Quantity One program (Bio-Rad, United States). The portion of the crRNA involved to the duplex formation was calculated as the retention of the complex intensity band to the sum of both intensities in the line. The dissociation constant was calculated by the formula

$$\ln(M_0 - \alpha \cdot RNA_0) = \ln(\alpha/(1-\alpha)) + K_d,$$

where M_0- initial concentration of PC-DNA, RNA_0 – initial concentration of crRNA, α - fraction of an oligonucleotide in a duplex, K_d - duplex dissociation constant. The results are presented in Table S3.

Table S3. Dissociation constants and melting temperatures of the duplexes of PC-DNA and their analogs with crRNA_Flu.

**Name**	**Sequences, 5'-3'**	**K_d, nmol/L**	**T_m**
crRNA		-	34,5±0,3
B20_c/crRNA	5'-TTTTTTACAAATTGAGTTATCC	109,3±9,4	40,8±0,2
B20_PL3/crRNA	5'-TTTTTT-PL-ACAAA-PL-TTGAG-PL-TTATCC-***Pyr***	n.d.	28,8±0,1
B30_c/crRNA	5'-GCTCTAAAACTTTTTTACAAATTGAGTTAT	13,8±3,4	52,8±0,1
B30_PL2_c/crRNA	5'-GCTCTAAAAC-PL-TTTTTTACAA-PL-ATTGAGTTAT	18,5±5,5	48,1±0,3
B30_PL3/crRNA	5′-GCTCTAAA-PL-ACTTTTT-PL-TACAAAT-PL-TGAGTTAT-***Pyr***	33,8±6,8	44,7±0,1

PL – photocleavable linker on the base of 1-(2-nitrophenyl)-1,2-ethandiol; ***Pyr*** – pyrene residue. crRNA_Flu - 5′-AUAACUCAAUUUGUAAAAAAGUUUUAGAGCUAUGCUGUUUUG-***Flu***; ***Flu*** – fluorescein residue.

Carbon encapsulated iron nanoparticles characterization

High-resolution TEM images were obtained using the JEM-2010 electron microscope (JEOL, Japan) with lattice-fringe resolution of 0.14 nm and accelerating voltage of 200 kV. The high resolution images of periodic structures were analyzed by the Fourier method. Local energy dispersive X-ray analysis (EDXA) was carried out using the EDX spectrometer (EDAX Co.), fitted with the Si (Li) detector, at resolution of 130 eV. The samples for the HRTEM study were prepared on a perforated carbon film mounted on a copper grid. XRD analysis was carried out using the Bruker D8 Advance diffractometer, equipped with the Lynxeye (1D) linear detector, over the angular range of 10–75º at 2Θ = 0.05º with the storage time of 1 s for each point. Monochromatic CuK-radiation (1.5418˚A) was applied in these experiments. Magnetic susceptibility was measured by the magnetometer SM-150L ZH Instrument, and SQUID magnetometer MPMSXL (Quantum Design).

The research results are given in [1]. For clarity, Figure S1A shows the morphology of the synthesized material. The corresponding nanoparticle size distribution function is shown in Figure S1B. The dependence of magnetization on the applied magnetic field is shown in Figure S1C. The absence of remanent magnetization indicates that the synthesized material is superparamagnetic.

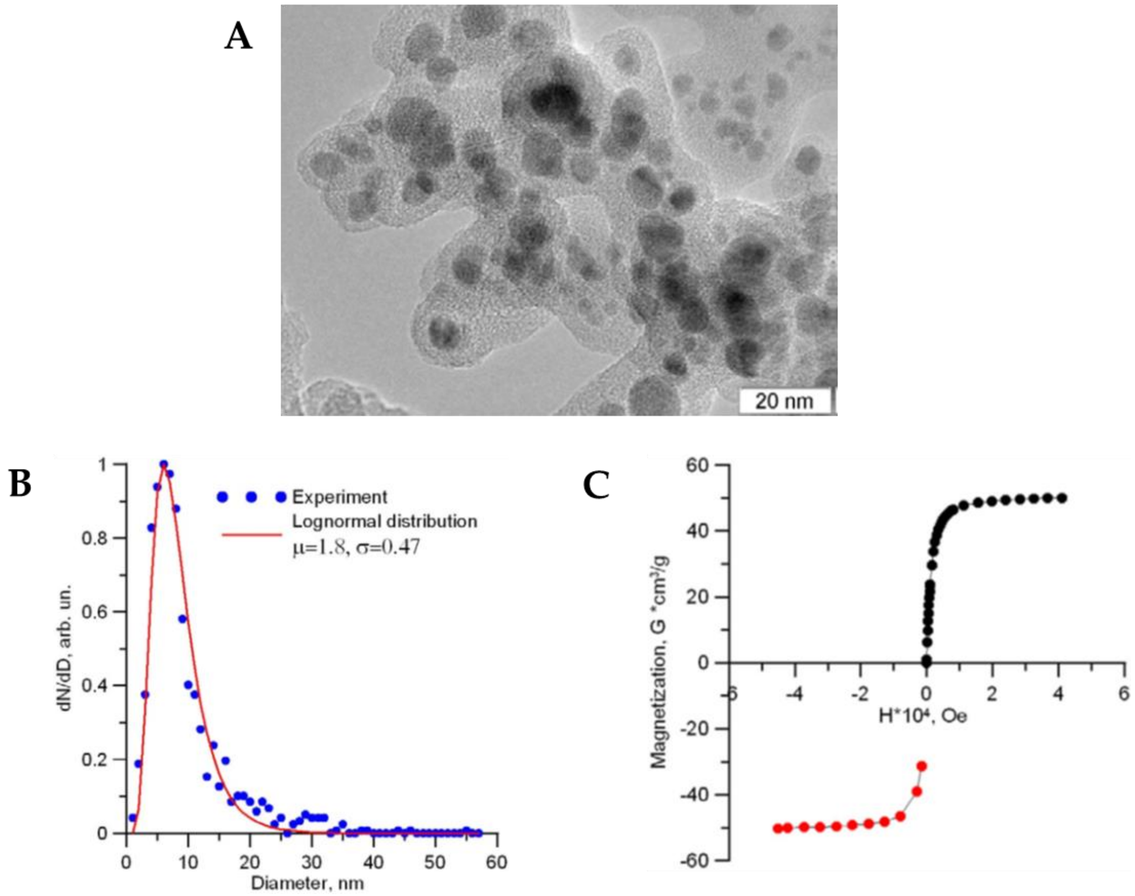

Figure S3. The CEINPs characterization. (A) The morphology of the synthesized material. (B) Size distribution function. (C) Magnetization of Fe-C soot.

References

1. Novopashin, S. A.; Serebryakova, M. A.; Zaikovskii, A. V. Morphology, Chemical Composition, and Magnetic Properties of Arc Discharge Fe–C Soot. In 3rd International Multidisciplinary Microscopy and Microanalysis Congress (InterM). Springer Proceedings in Physics. vol 186.; Oral, A., Bahsi Oral, Z., Eds.; Springer: Cham., 2016; pp. 9130–9133.
